# Identification and Characterisation of Putative Enhancer Elements in Mouse Embryonic Stem Cells

**DOI:** 10.1177/1177932220974623

**Published:** 2021-02-09

**Authors:** Anna Mantsoki, Karla Parussel, Anagha Joshi

**Affiliations:** 1 Division of Developmental Biology, The Roslin Institute, The University of Edinburgh, Midlothian, UK; 2 Computational Biology Unit, Department of Clinical Science, University of Bergen, Bergen, Norway

**Keywords:** epigenetics, enhancers, gene expression

## Abstract

Enhancer elements control mammalian transcription largely in a cell-type-specific manner. The genome-wide identification of enhancer elements and their activity status in a cellular context is therefore fundamental to understanding cell identity and function. We determined enhancer activity in mouse embryonic stem (ES) cells using chromatin modifications and characterised their global properties. Specifically, we first grouped enhancers into 5 groups using multiple H3K4me1, H3K27ac, and H3K27me3 modification data sets. Active enhancers (simultaneous presence of H3K4me1 and H3K27ac) were enriched for binding of pluripotency factors and were found near pluripotency-related genes. Although both H3K4me1-only and active enhancers were enriched for super-enhancers and a TATA box like motif, active enhancers were preferentially bound by RNA polII (s2) and were enriched for bidirectional transcription, while H3K4me1-only enhancers were enriched for RNA polII (8WG16) suggesting they were likely poised. Bivalent enhancers (simultaneous presence of H3K4me1 and H3K27me3) were preferentially in the vicinity of bivalent genes. They were enriched for binding of components of polycomb complex as well as Tcf3 and Oct4. Moreover, a ‘CTTTCTC’ de-novo motif was enriched at bivalent enhancers, previously identified at bivalent promoters in ES cells. Taken together, 3 histone modifications successfully demarcated active, bivalent, and poised enhancers with distinct sequence and binding features.

## Introduction

Embryonic stem (ES) cells have an indefinite proliferative life span and have a potential for both generating daughter cells with equivalent potential or cells ready for differentiation, that is, to establish unique gene expression profiles characteristic to different cell and tissue types during development. The delicate balance between self-renewal and the competency-to-differentiate, is achieved by epigenetic and transcription control. This process is tightly regulated by transcription factors and chromatin-associated proteins, which respond to signalling pathways which in turn responds to environmental cues.^1^ Epigenetic and transcription factors bind genomic regions proximal (promoters) and distal (enhancers) to the gene transcription start site (TSS) to control gene regulation. Collaborative efforts such as the FANTOM^2^ and Roadmap Epigenomics^3^ projects have now successfully built enhancer and promoter repertoires across hundreds of cell types with an estimated 1.4% of the human genome associated to putative promoters and about 13% to putative enhancers. The vast majority of disease susceptibility loci lie in non-coding genomic regions, particularly in enhancer regions^4,5^ and have been estimated to explain a greater proportion of the heritability for some disorders than variants in coding regions.^6,7^ Despite their known functional importance during development and disease, computational approaches towards genome-wide identification of enhancer elements remains a major challenge to date. This is due to various factors, including enhancers are typically distal to the TSS with no fixed distance preference, can range from a few to few hundred bases length and tend to be cell-type specific.^8^ On the other hand, regulatory regions (enhancers and promoters) have sequence features enriched for motifs of known transcription factors and evolutionarily conserved facilitate computational prediction. Moreover, enhancers can be distinguished from promoters using the presence of certain regulatory proteins including p300 (an acetyltransferase), BRG1 (a chromatin remodelled), the presence of histone modifications (H3K4me1 and H3K27ac), and unstable nucleosomes.^9^ Interestingly, several experiments have recently shown that enhancer regions get transcribed to form short, often bidirectional transcripts, called enhancers RNAs or eRNAs.^10^ Enhancer RNA expression is correlated with the expression of target genes, many a times in a stimulus-dependent manner and therefore can be used to predict enhancers and their target genes.^11^ Several functional mechanisms of gene regulation have been described in literature including enhancer-promoter looping, recruiting transcriptional machinery, and facilitating RNA polymerase pause-release.^12^ It is important to note that, eRNAs might not always be a predictor of enhancer activity or localisation, and not all functional enhancers have to be transcribed in eRNAs.

Enhancer regions have been classified into different groups based on their length, motif content, or chromatin status.^13^ Super-enhancers were defined as large genomic regions (>3 kb) near highly expressed genes, differing from typical enhancers in size, transcription factor density and content, ability to activate transcription, and sensitivity to perturbation.^14^ But, a CRISPR/Cas9-mediated deletion several super-enhancers clusters and isolated enhancers in mouse ES cells demonstrated enhancers and super-enhancers have an equivalent regulatory role in ES cells^15^ and iPS cells.^16^ The chromatin state is thought to be more functionally relevant with positive feedback loop between permissive chromatin and translation during early embryonic development.^17^ It is important to note that though histone modifications are widely used to define chromatin functional state, they are only predictions and not a proven fact. Furthermore, the experimental validations have demonstrated that they contain large number of false positives.^16,18^ Enhancers are marked by putative active (H3K4me1) or putative repressive (H3K27me3) chromatin modifications according to the cellular context. H3K4me3 (putative active) and H3K27me3 (putative repressive) modifications were found co-localised at promoters and enriched in developmentally regulated genes in mouse ES cells.^19^ Bivalent promoters were subsequently identified in human ES cells and were similarly enriched for developmental regulators^20^ and conserved their chromatin signature across species.^21^ The bivalent state is thought to assure silencing of key developmental controllers in ES cells while keeping them poised for activation during differentiation.^22^ Similar to promoters, putative enhancers marked with the bivalent enhancers are defined by the presence of the repressive mark H3K27me3 and active mark H3K4me1. Bivalent promoters identified in many mature lineages including T-cells,^23^ neural progenitor cells (NPCs), and mouse embryonic fibroblast (MEFs).^24^ Similarly, putative bivalent enhancers were identified in pro-B cells and adult liver,^25^ fibroblast-derived adipocytes, and bone-marrow macrophages.^26^ In summary, bivalent domains are present in enhancers and promoters and are thought to prime regulatory regions for their differentiation trajectory.^27^ An integrative analysis of bivalent promoters in mouse ES cells demonstrated that most H3K27me3 promoters were bivalent.^28^ Importantly, most bivalent promoters did not resolve into active or repressed chromatin in differentiated lineages, with many bivalent chromatin domains in fact remained bivalent across diverse cell types.^29^

Similarly, to study the functional relevance of chromatin signature of enhancer elements, we performed systematic analysis of putative enhancers in mouse ES cells by data integration. Specifically, we first grouped putative enhancers into 5 groups using multiple H3K4me1, H3K27ac, and H3K27me3 modification data sets. Putative active enhancers (simultaneous presence of H3K4me1 and H3K27ac) were enriched for binding of pluripotency factors and were found near pluripotency-related genes. Although both putative H3K4me1-only and active enhancers were enriched for super-enhancers and a TATA box like motif, putative active enhancers were preferentially bound by RNA polII s2 form and were enriched for bidirectional transcription, while putative H3K4me1-only enhancers were enriched for RNA polII (8WG16) suggesting they were likely poised. Putative bivalent enhancers (simultaneous presence of H3K4me1 and H3K27me3) were preferentially in the vicinity of bivalent genes. They were enriched for binding of components of polycomb complex as well as Tcf3 and Oct4. Moreover, a ‘CTTTCTC’ de-novo motif was enriched at putative bivalent enhancers, previously identified at bivalent promoters in ES cells.

## Material and Methods

### Data collection and processing

We collected ChIP sequencing raw data for H3K4me1, H3K27ac, and H3K27me3 profiles with 4 samples of each type in mouse ES cells from GEO^30^ and Roadmap Epigenomics.^31^ The accession numbers (containing the information about the ES cell lines and culture conditions) for raw data for each sample are provided in Supplemental Table S1. After mapping the reads to mm10 genome assembly, we called peaks in each sample using SICER.^32^ Input controls were not used, if they were not available for the samples. Specific parameters for running SICER for H3K4me3 and H3K27ac *window* = 200 and *gap size* = 200 and *f*or H3K27me3, *window* = 200 and *gap size* = 2 × 300, since H3K27me3 covers wider chromatin domains. The rest of the parameters (same for H3K4me3, H3K27ac, and H3K27me3) were *effective genome fraction =* 0.7, *redundancy threshold* = 1, *fragment size* = 150 and *E-value =* 100. We filtered out GENCODE M2 TSSs, which include both protein-coding and non-coding genes, to select enhancer peaks (Supplemental Table S1). Promoter regions were defined as 1 kb region around GENCODE TSSs.

### Putative enhancer classification

We created a genomic region by sample matrix by merging all peak files where ‘1’ represented a peak present in a given genomic region in a given sample and ‘0’ otherwise. Specifically, H3K4me1 peaks across diverse tissue and cell lines from ENCODE mouse data and 4 samples ES cell were merged using the following schema (Supplemental Table S2). The H3K4me1 peaks with an overlap of at least 25% with one of the peaks where the width of the merged peak was the union of 2 peaks. The merged list of H3K4me1 peaks was treated as ‘putative enhancers’ forming the rows of the matrix described above. The peaks for H3K4me1, H3K27ac, and H3K27me3 in ES cells overlapping at least 25% of the ‘putative enhancer’ peaks were considered overlapping and the H3K4me3 peak was defined as putative enhancer region. We then identified high confidence putative active enhancers by simultaneous presence of H3K4me1 and H3K27ac in 3 or more sample pairs and absence of H3K27me3 in all 4 samples and high confidence putative bivalent enhancers by simultaneous presence of H3K4me1 and H3K27me3 in 3 or more sample pairs and absence of H3K27ac in all 4 samples. We also defined H3K4me1-only, H3K27ac-only, and H3K27me3-only peaks by the presence of each chromatin modification in 3 or more samples and absence of other modifications. We have provided the genomic co-ordinates for all predicted enhancers in each enhancer group as Supplementary Material 2.

### Transcription and epigenetic control

To identify transcription and epigenetic factors enriched at putative bivalent, active, and H3K4me1-only enhancers, we used data from over 150 ChIP-seq experiments for transcription and epigenetic factors in mouse ES cells^33^ and the significance of overlap was calculated using a hypergeometric test. Enriched regions or peaks from ChIP sequencing data for epigenetic and transcription factors in mouse ES cells were downloaded from CODEX database.^33^ If the putative enhancer region from this study overlapped more than 50% with TF binding peaks from CODEX database, the peak was associated with the putative enhancer, that is, transcription factor was assumed to occupy that putative enhancer region. We collected ChIP sequencing data for 3 RNAPII modifications: RNAPIIS5P, RNAPIIS7P and 8WG16, PRC2 components: Suz12, Jarid2, and PRC1 subunits: Cbx7 and Ring1b in mouse ES cells and the enrichment of these factors at putative bivalent enhancers was calculated using BEDtools^34^ and density plots were visualised using *R*. The TSS sites from FANTOM5 were downloaded from http://fantom.gsc.riken.jp/5/data/.

### Enrichment analysis

The promoter regions in mouse ES cells grouped according to chromatin marks as bivalent, active (H3K4me3-only), H3K27me3-only, and latent (absence of both marks) were obtained from Mantsoki et al.^28^ The data for ES cell putative enhancers in 2i culture^35^ and ES cell putative super-enhancers^14^ was obtained from respective publications. The sequence motif enrichment analysis, the genomic location grouping (intergenic, intron, exon, TTS 3’ or 5’) as well as distance to the nearest TSS was calculated using HOMER.^36^ The de-novo motif analysis was performed using HOMER with default parameters. Mammalian conservation tracks were downloaded from UCSC genome browser and conservation status was assigned if more that 75% of region overlapped with mammalian conserved regions. We used a stringent threshold to select the enriched motifs (motif must be present in at least 10% of sequences and with a *P* value < 1e−10). Gene ontology functional analyses for the putative bivalent, active, and H3K4me1-only enhancers were done using DAVID.^37^ The TSSs predicted by the CAGE data as putative enhancers were obtained from the FANTOM consortium.^2^ The enrichments of overlaps were calculated using a hypergeometric test and *P* values were corrected using Bonferroni correction. The analysis was performed using *R* and shell scripts. All analysis was performed in mm10 genome assembly. The data sets provided by authors in mm9 were converted to mm10 using the liftover tool.^38^

### Putative enhancers in other tissues and during reprogramming

The peaks for the chromatin marks (H3K4me1, H3K27me3, and H3K27ac) across mouse cell lines and tissues were downloaded from the mouse ENCODE resource https://genome.ucsc.edu/encode/downloadsMouse.html (please refer to Supplemental Table S3 for details). The peaks for the chromatin marks (H3K4me1, H3K27me3, and H3K27ac) during reprogramming by 4 transcription factors Oct4, Sox2, Klf4, and Myc (OSKM) towards pluripotency were obtained from GEO, GSE67520.^39^

## Results

### Classification of putative enhancers in mouse ES cells by integrating ChIP-seq data

**Figure 1.**
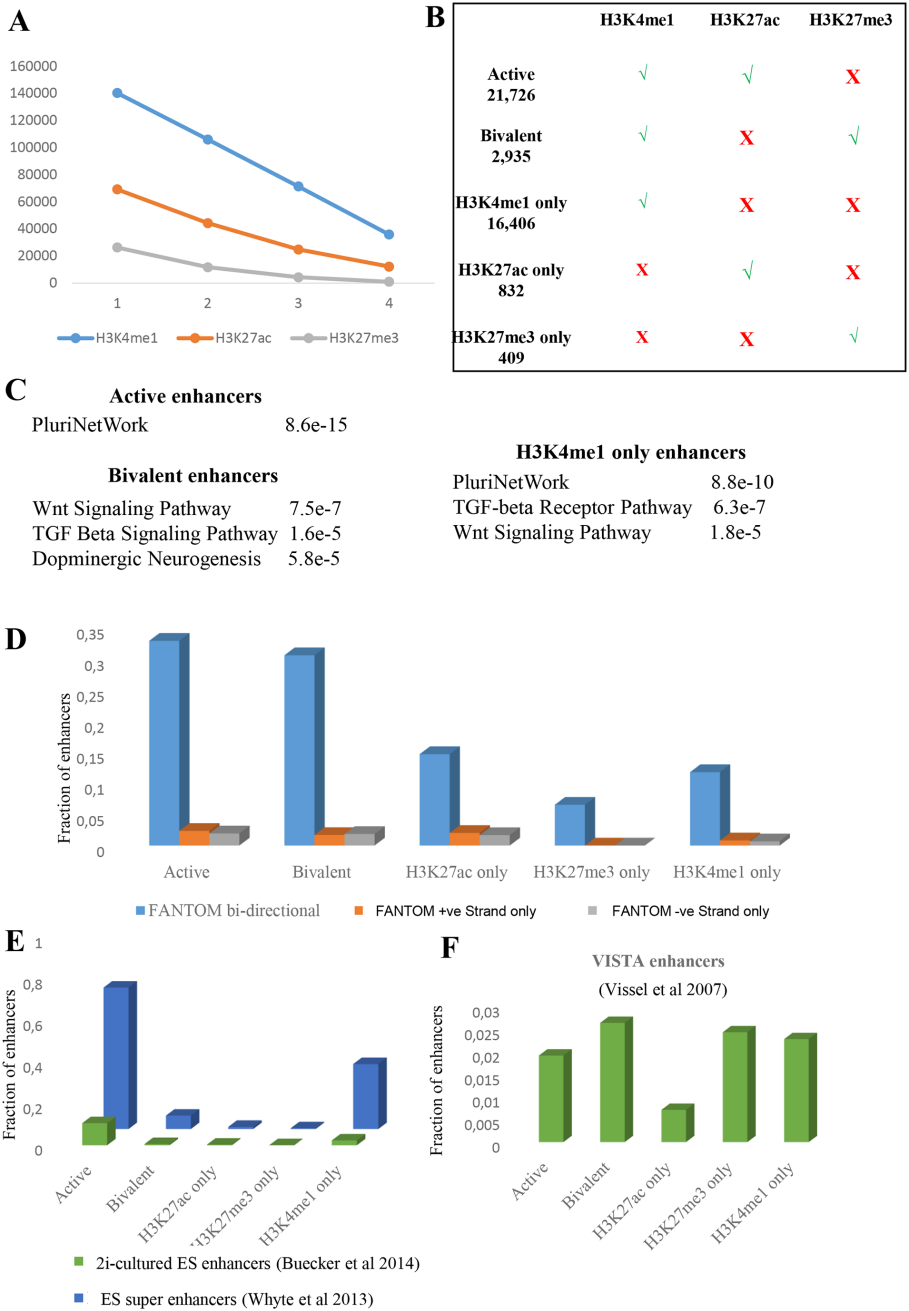
(A) The number of peaks in N (x axis) or more samples for each of the modification (H3K4me1 – red, H3K27ac – green, and H3K27me3–blue). (B) summary of enhancer groups based on the presence of histone modifications. (C) Gene ontology enrichment categories for the genes neighbouring bivalent, active and H3K4me1-only enhancers. (D) The fraction of transcription start sites in mouse ES cells identified by the FANTOM consortium overlapping with enhancer groups. (E) The fraction of enhancers in 2i- cultured ES cells and super-enhancers in ES cells overlapping with different enhancer groups. (F) The fraction of predicted by VISTA enhancer browser overlapping with different enhancer groups.

To identify and characterise putative enhancer elements in mouse ES cells, we collected ChIP sequencing data sets for H3K4me1, H3K27ac, and H3K27me3 modifications in mouse ES cells from independent studies (4 samples for each modification, Supplemental Table S1). Putative enhancer peaks in each sample were determined by removing the peaks identified using SICER^32^ overlapping with GENCODE^40^ promoters. This resulted into about 120 thousand nonpromoter genomic regions with H3K4me1 modifications in at least 1 of 4 samples (Figure 1A). About half of these regions were occupied by H3K27ac in at least 1 of the 4 samples and about 20% of these regions were occupied by H3K27me3 in at least 1 of the 4 samples. Importantly, H3K27ac and H3K27me3 tended to be mutually exclusive. For example, of more than 9000 peaks with H3K4me1 and H3K27ac modifications in all 8 samples, more than 8000 had no H3K27me3 in any of the samples. Nevertheless, over 200 peaks were marked with both H3K27ac and H3K27me3 in at least 3 samples. We classified 21 725 peaks with simultaneous presence of H3K4me1 and H3K27ac in 3 or more sample pairs and absence of H3K27me3 in all 4 samples as high confidence putative active enhancers and 2935 peaks with simultaneous presence of H3K4me1 and H3K27me3 in 3 or more sample pairs and absence of H3K27ac in all 4 samples as high confidence putative bivalent peaks. A total of 16 406 peaks present in 3 or more H3K4me1 samples and not in any of H3K27ac or H3K27me3 samples were called putative H3K4me-only enhancers. Similarly, 832 putative H3K27ac-only enhancers and 409 putative H3K27me3-only enhancers were defined (Figure 1B).

We mapped regulatory regions to the TSS of the nearest gene resulting into on average 2.5 regulatory regions mapped to a gene. We then calculated functional enrichment and pathway enrichments for each gene list (Figure 1C). Gene neighbouring ‘putative active enhancers’ were enriched for pluripotency network genes (*P* value < 8.6e−15) and genes neighbouring ‘putative bivalent enhancers’ were enriched for Wnt Signalling Pathway (*P* value < 7.5e−7) and TGF Beta Signalling Pathway (*P* value < 1.6e−5). Interestingly, genes neighbouring ‘putative H3K4me1-only enhancers’ were enriched for both pluripotency network genes (*P* value < 8.8e−10) and Wnt Signalling Pathway (*P* value < 6.3e−7) and TGF Beta Signalling Pathway (*P* value < 1.8e−5).

Andersson et al^41^ used bidirectional expression from CAGE data to determine putative enhancer regions across multiple human and mouse cell types. We collected 44 507 bidirectional TSSs as well as 51 807 (positive-strand) and 46 434 (negative-strand) unidirectional TSSs in ES cells from FANTOM5 resource.^2^ We noted that significantly higher fraction of regulatory regions in all groups overlapped with bidirectional TSSs compared to unidirectional TSSs (Figure 1D). We noted that about a third of both putative active and bivalent enhancers showed bidirectional expression or in other words, the majority of the regulatory regions defined as active or bivalent using chromatin modifications were not identified using bidirectional transcription signature.

Putative enhancers defined by published ChIP sequencing data of ES cells cultured in 2i medium condition^35^ showed a very small overlap with the enhancers in all 3 groups (Figure 1E). In contrast, putative ‘super-enhancers’ in ES cells^14^ were highly enriched in both active and H3K4me1-only marked enhancers (Figure 1E). We collected enhancers predicted across diverse tissues and cell types in mouse from VISTA enhancer database^42^ and noted that they showed a small overlap with all putative enhancer groups (Figure 1F). Notably, putative H3K27me3-only enhancers showed similar overlap as putative active and bivalent enhancers (Figure 1F). We further confirmed that predicted enhancers across tissues in fact had a small overlap with all possible putative enhancers regions in ES cells (over 140 K), suggesting most tissue-specific enhancers are likely latent (with no histone modification) in ES cells.

### Putative bivalent enhancers are enriched near bivalent gene promoters

**Figure 2.**
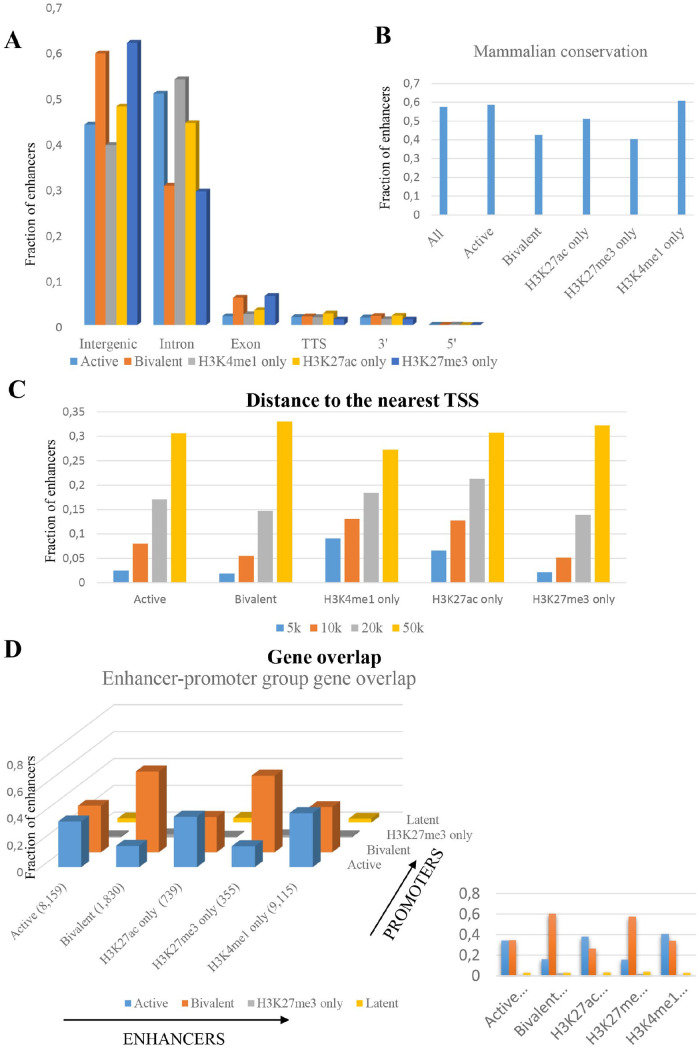
(A) The fraction each enhancer group in a specific genomic location (intergenic, intron, exon, 3’ and 5’) colour coded according to the enhancer group. (B) The fraction of each enhancer group overlapping with the mammalian conservation. (C) The fraction each enhancer group within a specific genomic distance window from the nearest TSS colour coded according to the distance window. (D) The overlap between the genes neighbouring the different promoter and enhancer groups representing bivalent enhancers and promoters are present near similar genes.

We further analysed whether specific genomic regions were preferred by putative enhancers. Most putative enhancers were present in either intergenic or intronic regions for all putative enhancer types (Figure 2A). Given that mammalian genome has far more intergenic than intronic sequence, introns were enriched for enhancer elements of all types. Specifically, putative active and H3K4me1-only enhancers were highly enriched for intronic regions (*P* value < 1e−50) while putative bivalent enhancers were enriched to a lesser extent (*P* value < 1e−3).

We downloaded genomic regions conserved across mammals from UCSC genome browser and noted that more than half of all putative enhancers were conserved across mammals. This fraction did not significantly change for any of the enhancer categories (Figure 2B).

To evaluate whether different putative enhancers showed a bias for specific genomic distance with respect to the nearest TSS. We calculated distance to the nearest TSS from each putative enhancer and noted that most enhancers were promoter distal with over half more than 50 kb away from the nearest TSS (Figure 2C). About 10% of H3K4me1-only enhancers formed an exception being within 5 kb of the nearest TSS.

We obtained the gene list likely to be regulated by putative enhancers in each category by mapping the putative enhancers to the TSS of the nearest gene (Supplemental Table S5). We have previously identified high-confidence bivalent, active (HK4me3 only), H3K4me3-only and latent (no mark) promoters in mouse ES cells.^28^ To test whether specific enhancer types are enriched near specific promoter types, we calculated the gene overlap between enhancer and promoter categories. Putative bivalent enhancers were highly enriched near bivalent promoters (Figure 2D). This postulated that bivalent promoters and enhancers might be part of a wider bivalent chromatin structure.

### Specific epigenetic and transcription factors are enriched at putative active and bivalent enhancers

**Figure 3.**
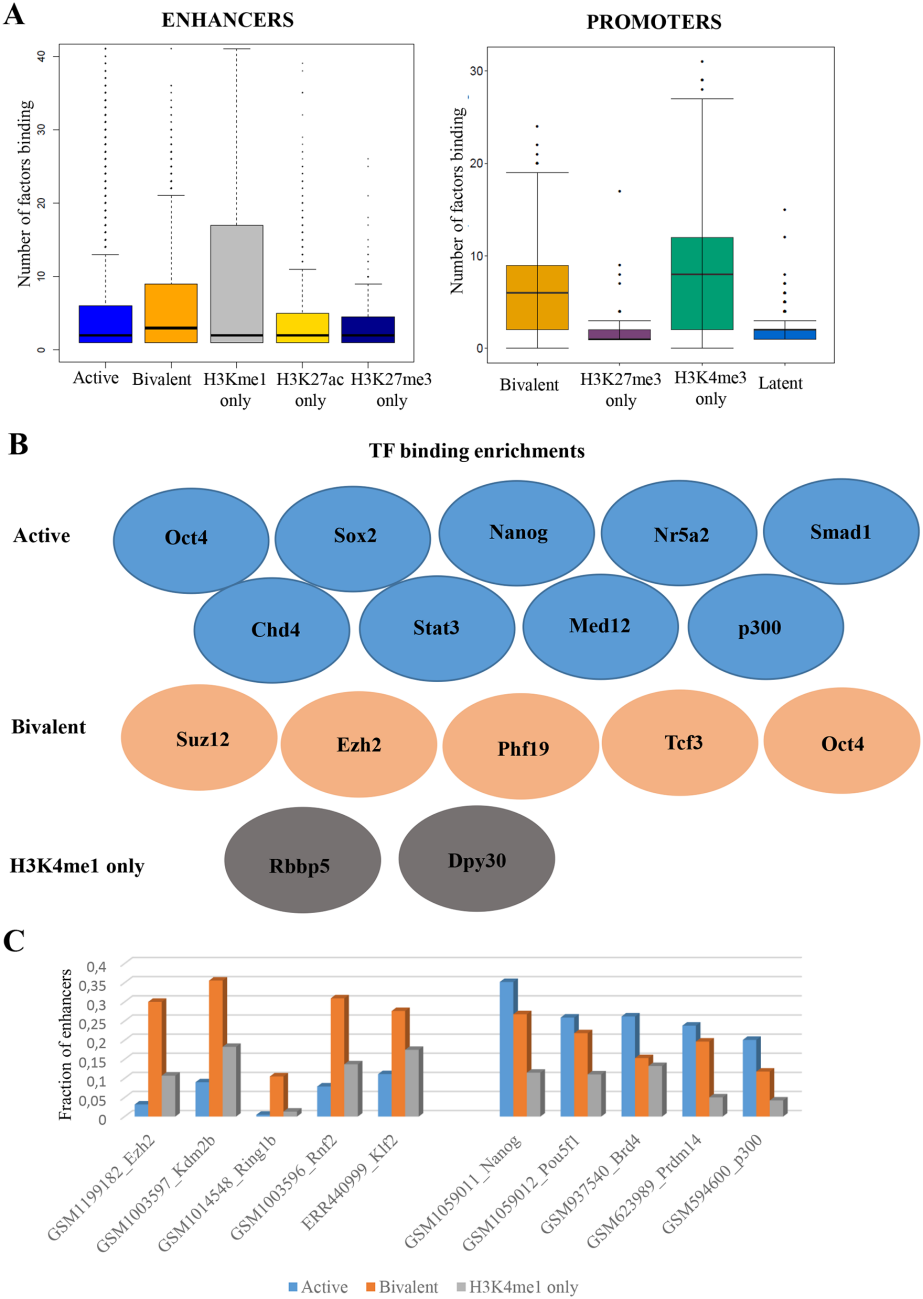
(A) The box plots of number of factors binding at different enhancer groups (left) and promoter groups (right) using CODEX database. (B) The epigenetic and transcription factors enriched at the active, bivalent, and H3K4me1-only enhancers. (C) The fraction of active, bivalent, and H3K4me1-only enhancers overlapping with peaks of different epigenetic and transcription factors. The factors at left are enriched in bivalent enhancers while the factors at right are enriched for the active enhancers.

Voigt et al^43^ proposed that bivalency in ES cells was maintained due to occupancy of fewer transcription factors, while at active promoters, the H3K27me3 modification is averted by a high density of active transcription factors. We collected ChIP sequencing data for more than 150 factors in mouse ES cells^33^ and noted that bivalent promoters were indeed occupied by fewer transcription factors compared to the H3K4me3-only or active promoters (Figure 3A, right). Interestingly though, we noted that more factors were bound at putative bivalent enhancers compared to putative active enhancers in mouse ES cells (Figure 3A, left). Both bivalent and active promoters were occupied by many more factors than putative bivalent and active enhancers.

Bivalent promoters belong to 2 distinct groups in mouse ES cells. The first class consists of domains where only PRC2 exists ( ‘PRC2-only’) and the second one, called PRC1-positive, where PRC2 domains are also occupied by PRC1. PRC1-positive, in contrast to PRC2-only, were more expansive bivalent regions, highly conserved, and consisted numerous developmental promoters.^44^ We collected ChIP sequencing data for epigenetic and RNAPII modifications known to distinguish bivalent promoter subtypes.^28^ Specifically, we grouped 2 RNAPII modifications: RNAPIIS5P and RNAPIIS7P, 8WG16 (an antibody recognising nonphosphorylated C-terminal domain), PRC2 components: Suz12, Jarid2, and PRC1 subunits: Cbx7 and Ring1b density at putative bivalent enhancers in mouse ES cells (Supplemental Figure S1). We noted that similar to promoters, a fraction of putative bivalent enhancers indeed showed a strong occupancy of polycomb and RNAPII. Never the less, these enhancers formed about 10% of putative bivalent enhancers (Supplemental Figure S1), unlike promoters which formed about half of bivalent promoters.

We further calculated enrichment for binding of individual factors at each putative enhancer group (Supplemental Table S6). Active enhancers were significantly enriched for p300 and core pluripotency factors including Oct4, Sox2, Nanog, Smad1, and Stat3. Importantly, these factors were not enriched at putative H3K4me1-only enhancers (Figure 3B). Putative H3K4me1-only enhancers were specifically enriched for Rbbp5 and Dpy30, the common regulatory components of MLL complex. Putative bivalent enhancers were enriched for polycomb components Suz12 and Ezh2. Interestingly, they were also enriched for 2 pluripotency factors Tcf3 and Oct4.

As the number of binding events vary greatly across factors affecting the enrichment, we calculated the fraction of peaks in each group occupied by a given factor. Ezh2, Kdm2b, Rnf2, and Klf2 were present at about a third of putative bivalent enhancers while Nanog, Pou5f1, Brd4, and Prdm14 were present at about a quarter of putative active enhancers (Figure 3C). P300, together with Utx and Mll4, facilitates conversion of inactive enhancers to active enhancers in ESCs.^45^ Interestingly, though p300 was enriched at putative active enhancers, it was present at the minority (less than 25%) of putative active enhancers. This can likely be due to the number of peaks called in p300 ChIP-seq sample (9429 peaks) compared with more than 21 000 predicted active enhancers.

### Sequence motif enrichment at putative enhancer groups

In the previous section, we noted that putative active and bivalent enhancers were enriched for different epigenetic and transcription controller. To investigate whether the binding enrichment of these factors is also reflected in sequence motif enrichments at putative active and bivalent enhancers, we calculated known and de-novo motif enrichment using HOMER.^36^ We selected motifs enriched with a *P* value less that 1e−10 and present in at least 10% of enhancers. Both putative active and H3K4me1-only enhancers were enriched for KLF, FLI, and ETS motifs. Importantly, the sequence motif for pluripotency factors enriched at putative active enhancers was not highly enriched. This suggests that KLF, FLI, and ETS motifs might facilitate binding of pluripotency factors to the enhancers. Indeed, simultaneous depletion of Klf2, Klf4, and Klf5 lead to ES cell differentiation.^46^ No known motif was found enriched at bivalent enhancers using stringent cut-offs defined above. The lack of enrichment of known pluripotency factor motifs at putative active enhancers and polycomb comples components at bivalent enhancers might be due to wider peaks called in histone modification data.

**Figure 4.**
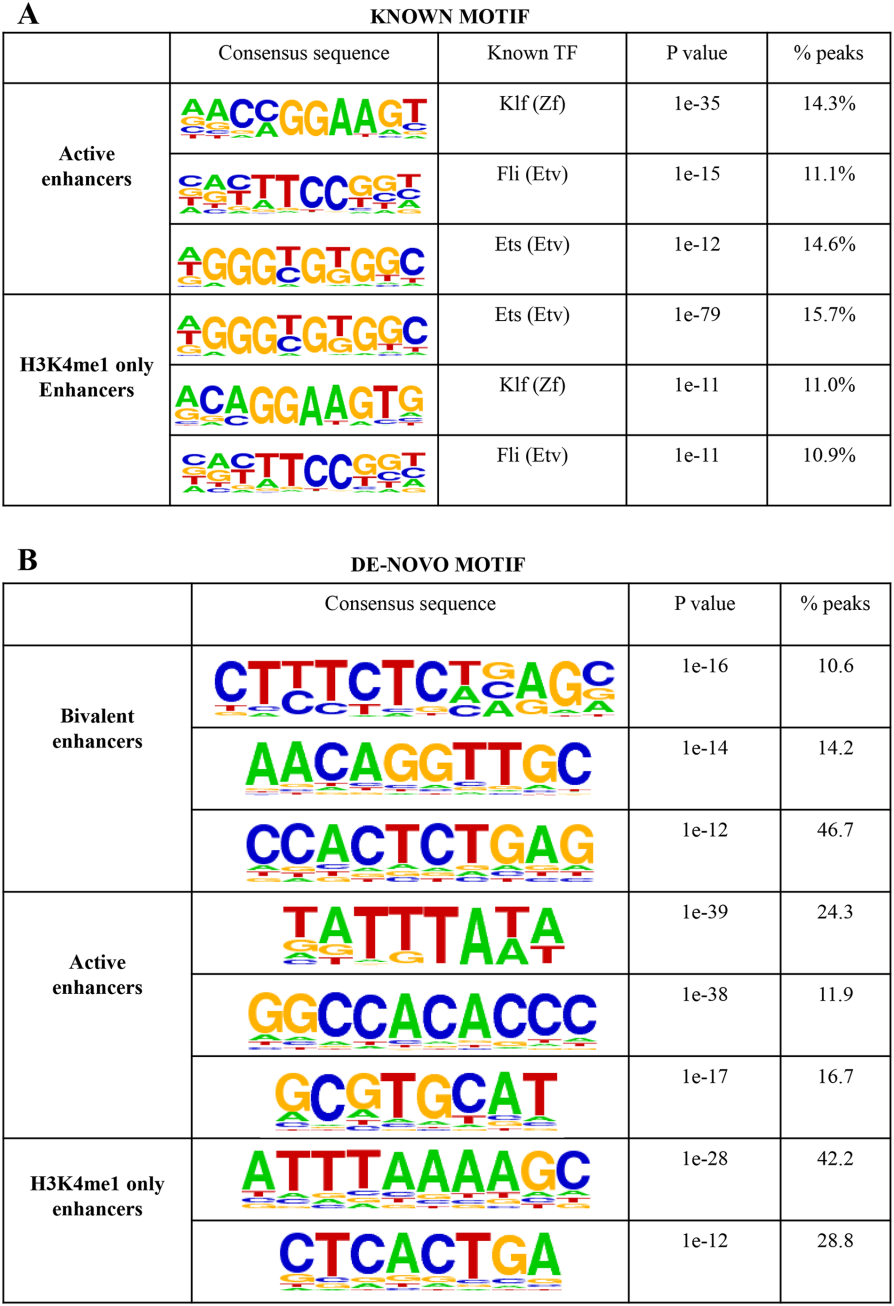
(A) Enriched known motifs at active and H3K4me1-only enhancers present in at least 10% of the enhancers identified by HOMER. (B) Enriched de-novo motifs at active and H3K4me1-only enhancers present in at least 10% of the enhancers identified by HOMER.

The de-novo motif search identified 3 motifs enriched in bivalent enhancers, 3 in putative active enhancers and 2 in putative H3K4me1-only enhancers (Figure 4B). The first de-novo motif identified at putative bivalent enhancers was previously identified enriched at bivalent promoters in ES cells.^28^ The second de-novo motif was similar to Myf6 from JASPAR database,^47^ while the third was present in about half of bivalent enhancers and was similar to Egr1 from JASPAR database.^47^ Of the 3 motifs in putative active enhancers, the first was similar to a POLII TATA box, the second similar to KLF motif while the third similar to MA0006.1, a bHLH motif from JASPAR database.^47^ Supporting known motif enrichments, the de-novo motif similar to TATA box was present in active and H3K4me1-only enhancers. Putative H3K4me1-only specific de-novo motif was similar to Zbtb3 motif from JASPAR database.^47^ As POLII TATA box like motif was enriched in putative active and H3K4me1-only enhancers, we calculated the overlap between enhancers and 3 forms of RNA POLII binding (s2, s5, and 8GW16) using ChIP sequencing data.^48^ Putative H3K4me1-only enhancers showed a much higher overlap with 8WG16 antibody, recognising nonphosphorylated Pol II while putative active enhancers showed a higher overlap for s2 antibody, specific for active, elongating Pol II (Supplemental Table S4).

### ES putative enhancer status in mouse tissues and during cellular reprogramming

**Figure 5.**
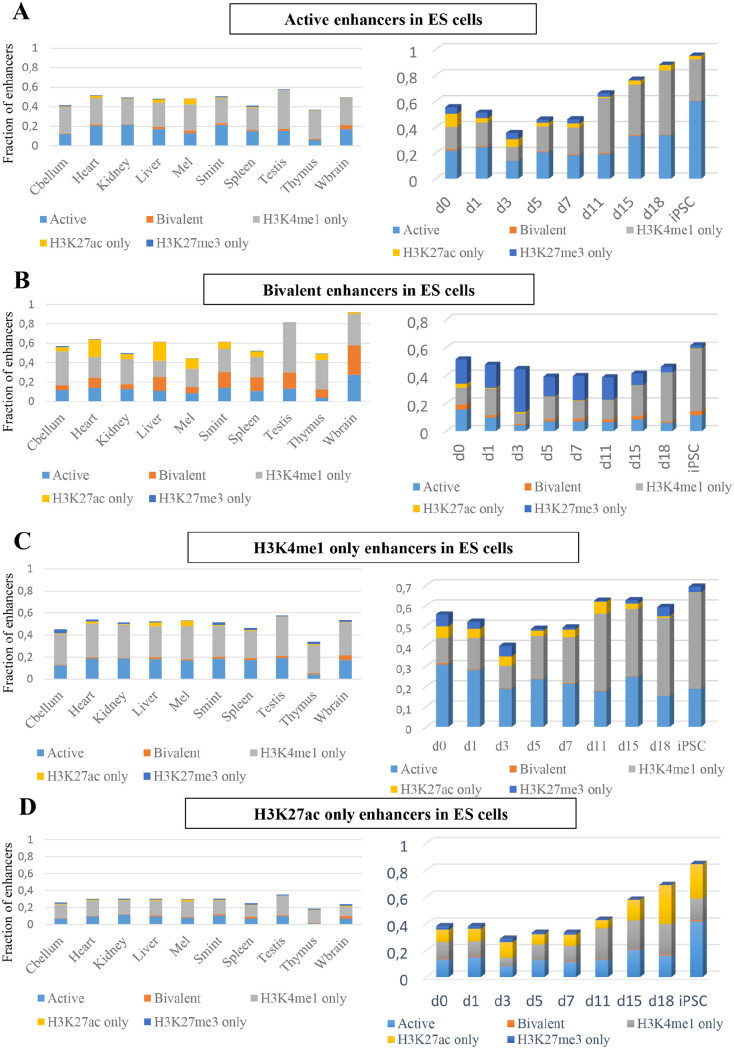
(A) The fraction of active enhancers overlapping with enhancer groups across different tissues from the mouse ENCODE (left) and with enhancers groups during cellular reprogramming (right) (Cbellum = cerebellum and Wbrain = whole brain). (B) The fraction of bivalent enhancers overlapping with enhancer groups across different tissues from the mouse ENCODE (left) and with enhancers groups during cellular reprogramming (right). (C) The fraction of H3K4me1-only enhancers overlapping with enhancer groups across different tissues from the mouse ENCODE (left) and with enhancers groups during cellular reprogramming (right). (D) The fraction of H3K27ac-only enhancers overlapping with enhancer groups across different tissues from the mouse ENCODE (left) and with enhancers groups during cellular reprogramming (right).

To study the dynamics of the 5 groups of putative enhancers in ES cells through differentiation, we collected H3K4me1, H3K27ac, H3K27me3 data for 10 tissues and cell types from the mouse ENCODE resource. Similar to the analysis of ES cells data, we classified the putative enhancers in each cell type in the same 5 groups, namely active, bivalent, H3K4me1-only, H3K27ac-only, and H3K27me3-only. We then calculated the overlap of each groups of putative enhancers in ES cells with all 5 enhancers groups in each of the 10 tissue and cell types individually (Figure 5, left). Putative active and H3K4me1-only enhancers in ES cells were mostly active or H3K4me1-only in other tissue of cell types (Figure 5A, C left). Putative bivalent enhancers in ES cells, though were predominantly H3K4me1-only in other tissue and cell types, about 10% retained bivalency across other tissue and cell types (Figure 5B, left). Interestingly, about 20% of putative bivalent enhancers in ES cells were H3K27ac-only in heart and liver (Figure 5B, left).

To explore how the above-noted patterns might establish during differentiation, we collected time-series data during cellular reprogramming by induced expression of 4 transcription factors Oct4, Sox2, Klf4, and Myc (OSKM) pushing somatic cell reprogramming towards pluripotency.^39^ Similar to the observation in other tissues and cell types, putative active and H3K4me1-only enhancers in ES cells were mostly active or H3K4me1-only during reprogramming. As expected putative active enhancers in ES cells showed a high overlap with putative active enhancers in iPSCs and putative H3K4me1-only enhancers also behaved similarly. Interestingly, putative bivalent enhancers in ES cells lost H3K27me3 and gained H3K4me1 during reprogramming.

## Discussion

Understanding enhancer function is fundamental to understanding cell-type-specific gene control. Here, we have defined putative enhancers based on the presence of H3K27ac, H3K27me3, and H3K4me1 modifications and characterised them in detail. We defined putative active enhancers by simultaneous presence of H3K4me1 and H3K27ac. We note that experimental validations of putative active enhancer predictions demonstrate that they contain high rate of false positives^16,18^ and have led to other definitions of active enhancers but there is no agreed consensus. Simultaneous presence of H3K4me1 and H3K27ac remains a common criterion used for identification putative active enhancers. We note that in this study used only histone modification data, as the aim was to identify high confidence (relatively wide) regulatory regions and not identification of precise regulatory site within them. In latter case, one would ideally integrate other data including p300 binding or nucleosome depletion measured via ATAC-Seq or DNAse hypersensitivity assays. Furthermore, we mapped the regulatory regions to the nearest TSS. Although this is the most widely used approach to map putative targets of regulatory regions, it is important to note that the nearest neighbour gene is not always the enhancer target. There are many approaches to improve the mapping including chromatin capture assays to computational models.^49^

Putative active enhancers showed a high overlap with FANTOM bidirectional putative enhancers, ES putative super-enhancers, and binding of many pluripotency factors. We note that the FANTOM bidirectional putative enhancers were identified across diverse tissue and cell types and therefore limiting the overlap. The properties of active enhancers were distinct from putative H3K4me1-only enhancers corroborating that the presence of H3K27ac is sufficient to distinguish putative active enhancers.^25^ The higher overlap of putative H3K4me1-only enhancers with RNA POLII 8WG16 antibody, with no enrichment for bidirectional expression points out that they might be poised. Intriguingly, genes near H3K4me1-only enhancers were also enriched for pluripotency-related genes. Furthermore, genes neighbouring ‘putative active enhancers’ were enriched for pluripotency network genes (*P* value < 8.6e−15) and genes neighbouring ‘putative bivalent enhancers’ were enriched for Wnt Signalling Pathway (*P* value < 7.5e−7), and TGF Beta Signalling Pathway (*P* value < 1.6e−5). Interestingly, genes neighbouring ‘putative H3K4me1-only enhancers’ were enriched for both pluripotency network genes (*P* value < 8.8e−10) and Wnt Signalling Pathway (*P* value < 6.3e−7) and TGF Beta Signalling Pathway (*P* value < 1.8e−5). We noted that putative H3K4me1-only enhancers share the (nearest) genes with putative active and bivalent enhancers, resulting into enrichment for the signalling pathways. H3K4me1 modification has sharp ChIP-seq signal compared to both H3K27ac and H3K27me3 modifications and therefore more consistent peak detection across data sets. One of the possibilities therefore is that at least some of the putative H3K4me1-only enhancers were in-fact putative active (missing H3K27ac) or bivalent enhancers (missing H3K27me3). It is also worth noting that bivalent enhancers had all 3 RNA Pol II peaks present (Supplemental Table S4).

We defined putative bivalent enhancers by simultaneous presence of H3K4me1 and H3K27me3 modifications. Putative bivalent enhancers were present near bivalent promoters and many maintained bivalency through differentiation. Other definitions of putative bivalent enhancers include simultaneous presence of H3K27ac and DNA methylation.^50^ Bivalent regions selected by this definition show a high overlap with our ‘putative active enhancers’ as most H3K27ac marked enhancers also carry H3K4me1 modification. Finally, we also identified a ‘CTTTCTC’ de-novo motif enriched at bivalent enhancers previously identified at bivalent promoters.

## Supplemental Material

sj-pdf-1-bbi-10.1177_1177932220974623 – Supplemental material for Identification and Characterisation of Putative Enhancer Elements in Mouse Embryonic Stem Cells Click here for additional data file. Supplemental material, sj-pdf-1-bbi-10.1177_1177932220974623 for Identification and Characterisation of Putative Enhancer Elements in Mouse Embryonic Stem Cells by Anna Mantsoki, Karla Parussel and Anagha Joshi in Bioinformatics and Biology Insights
