# Supplementary material for: Identification and Characterisation of Putative Enhancer Elements in Mouse Embryonic Stem Cells
Source: Bioinform Biol Insights. 2021 Feb 9;15:1177932220974623. doi: 10.1177/1177932220974623 (PMC7876754; doi:10.1177/1177932220974623)
Supplement: sj-pdf-1-bbi-10.1177_1177932220974623 – Supplemental material for Identification and Characterisation of Putative Enhancer Elements in Mouse Embryonic Stem Cells [file sj-pdf-1-bbi-10.1177_1177932220974623.pdf]

Supplementary material

for

Identification and characterisation of enhancer elements in mouse embryonic stem cells

Anna Mantsoki[1], Karla Parussel[1], and Anagha Joshi[1,*]

[1] Division of Developmental Biology, The Roslin Institute, The University of Edinburgh, Easter Bush, Midlothian, Eh25 9RG, UK.

Table 1: Table summarising chromatin modification datasets collected from public repositories with the histone modification, the public accession number and number of peaks identified in each sample.

Histone modification	Sample number	Number of Peaks
H3K4me1	SRR317224	80464
H3K4me1	SRR414939	101930
H3K4me1	SRR900935	116867
H3K4me1	SRR900936	54310
H3K27me3	SRR317225	24590
H3K27me3	SRR414936	27396
H3K27me3	SRR900947	49855
H3K27me3	SRR900948	48692
H3K27ac	GSE31039	16019
H3K27ac	GSE38596	3718
H3K27ac	GSE47949 (rep 2)	15853
H3K27ac	GSE47949 (rep 3)	7788

Table 2: Table summarising H3K4me1 chromatin modification datasets collected from the ENCODE to generate putative mouse enhancers, along with their accession number and lab generating the sample.

ENCSR000ADK	ES-E14	Michael Snyder, Stanford

ENCSR000ADQ	MEL cell line	Michael Snyder, Stanford
ENCSR000ADY	MEL cell line	Michael Snyder, Stanford
ENCSR000CAE	heart	Bing Ren, UCSD
ENCSR000CAF	kidney	Bing Ren, UCSD
ENCSR000CAG	bone marrow	Bing Ren, UCSD
ENCSR000CAI	cortical plate	Bing Ren, UCSD
ENCSR000CAL	cerebellum	Bing Ren, UCSD
ENCSR000CAO	liver	Bing Ren, UCSD
ENCSR000CAQ	lung	Bing Ren, UCSD
ENCSR000CAZ	embryonic fibroblast	Bing Ren, UCSD
ENCSR000CBC	spleen	Bing Ren, UCSD
ENCSR000CBF	ES-Bruce4	Bing Ren, UCSD
ENCSR000CCF	olfactory bulb	Bing Ren, UCSD
ENCSR000CCI	thymus	Bing Ren, UCSD
ENCSR000CCR	small intestine	Bing Ren, UCSD
ENCSR000CCV	testis	Bing Ren, UCSD
ENCSR000CDL	heart	Bing Ren, UCSD
ENCSR000CDP	placenta	Bing Ren, UCSD
ENCSR000CDW	liver	Bing Ren, UCSD
ENCSR000CEW	MEL cell line	Bing Ren, UCSD
ENCSR000CFA	brown adipose tissue	Bing Ren, UCSD
ENCSR000CFE	bone marrow macrophage	Bing Ren, UCSD
ENCSR000CGH	CH12.LX	Bing Ren, UCSD
ENCSR000CGN	ES-E14	Bing Ren, UCSD
ENCSR000DHK	erythroblast	Ross Hardison, PennState
ENCSR000DHQ	CH12.LX	Ross Hardison, PennState

ENCSR000DHR	megakaryocyte	Ross Hardison, PennState
ENCSR000DIY	G1E-ER4	Ross Hardison, PennState
ENCSR000DJB	G1E	Ross Hardison, PennState
ENCSR014MXQ	hindbrain	Bing Ren, UCSD
ENCSR032JUI	E14TG2a.4	Ross Hardison, PennState
ENCSR037HLB	midbrain	Bing Ren, UCSD
ENCSR045NOD	liver	Bing Ren, UCSD
ENCSR051CUH	intestine	Bing Ren, UCSD
ENCSR080GQM	heart	Bing Ren, UCSD
ENCSR091WNX	embryonic facial prominence	Bing Ren, UCSD
ENCSR118TPX	inflammation-experienced regulatory T-cells	Christina Leslie, MSKCC
ENCSR133EGP	liver	Bing Ren, UCSD
ENCSR141ZQF	forebrain	Bing Ren, UCSD
ENCSR157IVC	midbrain	Bing Ren, UCSD
ENCSR157LYR	intestine	Bing Ren, UCSD
ENCSR159RVN	intestine	Bing Ren, UCSD
ENCSR196ENU	kidney	Bing Ren, UCSD
ENCSR234ISO	liver	Bing Ren, UCSD
ENCSR238SGC	limb	Bing Ren, UCSD
ENCSR243JOL	forebrain	Bing Ren, UCSD
ENCSR251LFV	heart	Bing Ren, UCSD
ENCSR253IEG	midbrain	Bing Ren, UCSD
ENCSR257JSX	hindbrain	Bing Ren, UCSD
ENCSR258AED	embryonic facial prominence	Bing Ren, UCSD
ENCSR263CKR	neural tube	Bing Ren, UCSD
ENCSR272GNQ	midbrain	Bing Ren, UCSD

ENCSR308GFM	liver	Bing Ren, UCSD
ENCSR335WME	stomach	Bing Ren, UCSD
ENCSR339LMJ	heart	Bing Ren, UCSD
ENCSR349NIS	hindbrain	Bing Ren, UCSD
ENCSR364HOR	activated regulatory T-cells	Christina Leslie, MSKCC
ENCSR387YSD	lung	Bing Ren, UCSD
ENCSR391WSS	midbrain	Bing Ren, UCSD
ENCSR417TXZ	hindbrain	Bing Ren, UCSD
ENCSR419MSI	liver	Bing Ren, UCSD
ENCSR426EZM	limb	Bing Ren, UCSD
ENCSR436FYE	kidney	Bing Ren, UCSD
ENCSR442RYY	heart	Bing Ren, UCSD
ENCSR448TTC	neural tube	Bing Ren, UCSD
ENCSR449EUZ	midbrain	Bing Ren, UCSD
ENCSR450ITF	midbrain	Bing Ren, UCSD
ENCSR465PLB	forebrain	Bing Ren, UCSD
ENCSR487OLC	liver	Bing Ren, UCSD
ENCSR488OCA	heart	Bing Ren, UCSD
ENCSR496TBX	forebrain	Bing Ren, UCSD
ENCSR511WPK	embryonic facial prominence	Bing Ren, UCSD
ENCSR523IIH	lung	Bing Ren, UCSD
ENCSR529ERN	limb	Bing Ren, UCSD
ENCSR548BCO	limb	Bing Ren, UCSD
ENCSR548BKP	stomach	Bing Ren, UCSD
ENCSR556ZUY	forebrain	Bing Ren, UCSD
ENCSR608QBO	hindbrain	Bing Ren, UCSD

ENCSR617VBE	hindbrain	Bing Ren, UCSD
ENCSR645ETR	embryonic facial prominence	Bing Ren, UCSD
ENCSR663VWL	heart	Bing Ren, UCSD
ENCSR674PZU	kidney	Bing Ren, UCSD
ENCSR678FIT	midbrain	Bing Ren, UCSD
ENCSR695FPP	hindbrain	Bing Ren, UCSD
ENCSR751AAB	limb	Bing Ren, UCSD
ENCSR770OXU	liver	Bing Ren, UCSD
ENCSR782DGO	heart	Bing Ren, UCSD
ENCSR810EOA	limb	Bing Ren, UCSD
ENCSR825OWH	lung	Bing Ren, UCSD
ENCSR826TJQ	neural tube	Bing Ren, UCSD
ENCSR829YGD	intestine	Bing Ren, UCSD
ENCSR858AUB	lung	Bing Ren, UCSD
ENCSR874WRY	regulatory T cell	Christina Leslie, MSKCC
ENCSR875KRK	forebrain	Bing Ren, UCSD
ENCSR883ASH	kidney	Bing Ren, UCSD
ENCSR886IHN	heart	Bing Ren, UCSD
ENCSR907CPZ	stomach	Bing Ren, UCSD
ENCSR914QGB	embryonic facial prominence	Bing Ren, UCSD
ENCSR918ZSJ	neural tube	Bing Ren, UCSD
ENCSR921ILW	hindbrain	Bing Ren, UCSD
ENCSR940CMI	stomach	Bing Ren, UCSD
ENCSR972WEN	embryonic facial prominence	Bing Ren, UCSD
ENCSR975QSF	forebrain	Bing Ren, UCSD
ENCSR977DCO	neural tube	Bing Ren, UCSD

ENCSR992SHK	forebrain	Bing Ren, UCSD

Table 3: Table summarising chromatin modification datasets collected from the ENCODE across other cell and tissue types.

wgEncodeLicrHistoneCbellumH3k27acMAdult8wksC57bl6StdPk.broadPeak
wgEncodeLicrHistoneCbellumH3k27me3MAdult8wksC57bl6StdPk.broadPeak
wgEncodeLicrHistoneCbellumH3k4me1MAdult8wksC57bl6StdPk.broadPeak
wgEncodeLicrHistoneHeartH3k27acMAdult8wksC57bl6StdPk.broadPeak
wgEncodeLicrHistoneHeartH3k27me3MAdult8wksC57bl6StdPk.broadPeak
wgEncodeLicrHistoneHeartH3k4me1MAdult8wksC57bl6StdPk.broadPeak
wgEncodeLicrHistoneKidneyH3k27acMAdult8wksC57bl6StdPk.broadPeak
wgEncodeLicrHistoneKidneyH3k27me3MAdult8wksC57bl6StdPk.broadPeak
wgEncodeLicrHistoneKidneyH3k4me1MAdult8wksC57bl6StdPk.broadPeak
wgEncodeLicrHistoneLiverH3k27acMAdult8wksC57bl6StdPk.broadPeak
wgEncodeLicrHistoneLiverH3k27me3MAdult8wksC57bl6StdPk.broadPeak
wgEncodeLicrHistoneLiverH3k4me1MAdult8wksC57bl6StdPk.broadPeak
wgEncodeLicrHistoneMelH3k04me1MImmortalC57bl6StdPk.broadPeak
wgEncodeLicrHistoneMelH3k27acMImmortalC57bl6StdPk.broadPeak
wgEncodeLicrHistoneMelH3k27me3MImmortalC57bl6StdPk.broadPeak
wgEncodeLicrHistoneSmintH3k04me1MAdult8wksC57bl6StdPk.broadPeak
wgEncodeLicrHistoneSmintH3k27acMAdult8wksC57bl6StdPk.broadPeak
wgEncodeLicrHistoneSmintH3k27me3MAdult8wksC57bl6StdPk.broadPeak
wgEncodeLicrHistoneSpleenH3k27acMAdult8wksC57bl6StdPk.broadPeak
wgEncodeLicrHistoneSpleenH3k27me3MAdlt8wC57bl6StdPk.broadPeak
wgEncodeLicrHistoneSpleenH3k4me1MAdult8wksC57bl6StdPk.broadPeak
wgEncodeLicrHistoneTestisH3k04me1MAdult8wksC57bl6StdPk.broadPeak
wgEncodeLicrHistoneTestisH3k27acMAdult8wksC57bl6StdPk.broadPeak

wgEncodeLicrHistoneTestisH3k27me3MAdlt8wC57bl6StdPk.broadPeak
wgEncodeLicrHistoneThymusH3k04me1MAdult8wksC57bl6StdPk.broadPeak
wgEncodeLicrHistoneThymusH3k27acMAdult8wksC57bl6StdPk.broadPeak
wgEncodeLicrHistoneThymusH3k27me3MAdlt8wC57bl6StdPk.broadPeak
wgEncodeLicrHistoneWbrainH3k04me1UE14halfC57bl6StdPk.broadPeak
wgEncodeLicrHistoneWbrainH3k27acUE14halfC57bl6StdPk.broadPeak
wgEncodeLicrHistoneWbrainH3k27me3ME14halfC57bl6StdPk.broadPeak

Table 4: The table calculating overlap of enhancer peaks in three groups with three forms of RNA polymerase II binding in mouse ES cells

RNA polymerase II – ChIP sequencing	Active enhancers (21,726)	H3K4me1-only enhancers (16,406)	Bivalent enhancers (2,935)
GSM1059013_Polr2a 8WG16 (10587)	1295	2596	174
GSM1059013_Polr2a s2 (1956)	351	171	31
GSM1059013_Polr2a s5 (15110)	2858	2830	314

Table 5: The overlap of genes near different enhancer and promoter groups.

Promoter >	Total	Bivalent	Active	H3K27me3 only	Latent
Enhancer ˅		4,965	6082	391	30,012
Active (8,159)	8,159	1,410	1,699	21	757
Bivalent (1,830)	1,830	549	178	14	168
H3K27ac only (739)	739	97	170	1	77
H3K27me3 only (355)	355	101	34	2	43
H3K4me1 only (9,115)	9,115	1,532	2,246	23	780

Table 6: Enrichment (P value) calculated by a hypergeometric test for binding of (ChIP sequencing) over 150 factors in ES cells

factor	Bivalent	H3K27ac only	H3K27me3 only	H3K4me1 only	Active
GSM1014542_Ezh2	0	1	0.999712	1	1
GSM1014546_Kdm2b	1	1	1	0.970858	1
GSM1014548_Ring1b	2.17E-10	1	0.985919	1	1
GSM1015512_Gata4	1	1	1	1	1
GSM1015513_Gata4	1	0.999997	0.999993	1	1
GSM1041372_Ring1B	5.25E-06	0.999999	0.999734	1	1
GSM1041373_Cbx7	0.970546	0.978979	0.974368	1	1
GSM1058995_Kdm4b	1	0.032838	1	1	0
GSM1058996_Kdm4c	1	1	1	1	1
GSM1059004_Ezh2	2.43E-10	0.999999	0.996758	1	1
GSM1059011_Nanog	1	0.999938	1	1	0
GSM1059012_Pou5f1	1	0.999138	1	1	0
GSM1059024_Pou5f1	1	0.993793	1	1	0
GSM1059025_Ctr9	1	0.554428	0.999539	1	0.999977
GSM1059031_Pou5f1	1	0.999994	1	1	0
GSM1059032_Nanog	1	0.988926	1	1	0
GSM1065517_bCatenin	0.100947	0.068588	0.998636	1	0
GSM1065518_bCatenin	0.038724	0.157827	0.999906	1	0
GSM1169006_Jarid2	1.44E-07	0.999995	0.999929	1	1
GSM1169007_Jarid2	0.000888	1	0.998626	1	1
GSM1169010_Rest	1	1	0.996825	1	1
GSM1169014_Rnf2	0	1	0.999992	1	1
GSM1169017_Rnf2	1.01E-07	1	0.988764	1	1

GSM1184045_Nanog	0.999997	0.967715	1	1	0
GSM1199182_Ezh2	0	1	1	1	1
GSM1215218_Ezh2	0.268011	0.999997	0.999982	1	1
GSM288345_Nanog	3.55E-06	0.75672	0.980709	1	0
GSM288346_Oct4	0.000257	0.759093	0.956318	1	0
GSM288349_E2f1	1	1	1	0	3.84E-07
GSM288351_CTCF	1	1	1	1	1
GSM288354_Klf4	1	0.981417	0.999998	0.951564	0
GSM288355_Esrrb	1	0.813558	1	1	0
GSM288357_n-Myc	1	0.999783	0.999953	0	1
GSM307137_Oct4	0.998473	0.392075	1	1	0
GSM307140_Nanog	0.99313	0.866507	0.999998	1	0
GSM327668_Ezh2	0.88995	0.999813	0.998985	1	1
GSM415050_Mtf2	7.96E-06	0.999999	0.999702	1	1
GSM440256_Eset	0.999945	0.879949	0.450236	1	1
GSM515666_Ctr9	1	0.831978	0.983053	0.999992	0.997508
GSM560341_Smc1	0.99968	0.963279	0.169592	1	1
GSM560343_Smc3	1	0.999136	0.165322	1	1
GSM560345_Med12	1	0.878848	0.998893	1	0
GSM560346_Med12	1	0.615095	0.999919	1	0
GSM560347_Med1	1	0.385403	0.947181	1	0
GSM560350_Nipbl	0.999953	0.410067	0.681791	0.219815	0.00567
GSM560352_Ctcf	1	1	0.999754	1	1
GSM566277_Oct4	2.17E-05	0.969162	0.926825	1	0
GSM566278_Rbbp5	1	0.999887	0.991057	0	1
GSM591469_Rad21	1	0.999997	0.909467	1	1

GSM591470_Rad21	1	0.999994	0.989932	1	1
GSM594600_p300	1	0.584717	1	1	0
GSM611196_Sin3A	1	0.999998	0.999564	0	1
GSM611197_Sin3A	1	0.999962	0.977514	0	1
GSM623989_Prdm14	1	0.999809	1	1	0
GSM634247_Ctcf-Gfp	1	1	0.999538	1	1
GSM651192_Dpy30	0.999998	0.751655	0.855561	0	1
GSM656523_Ring1b	0.136248	0.995138	0.996938	1	1
GSM699164_p300	0.986563	0.376047	0.783801	1	0
GSM699165_CTCF	1	1	1	1	1
GSM700555_Phf19	0.317587	0.993897	0.99965	1	1
GSM700556_Phf19	0	1	0.997997	1	1
GSM749809_Ell2	1	0.93752	0.996451	1	0.004996
GSM749810_Aff4	1	0.997806	0.999974	1	0.022146
GSM774946_POL2	1	1	0.999995	0	1
GSM898372_Kdm6b	1	1	1	1	1
GSM905445_Ezh2	0.295181	1	0.999567	1	1
GSM915092_Ncoa3	0.97349	0.540037	0.589473	1	0
ERR440998_Klf2	1	1	1	6.49E-13	1
ERR440999_Klf2	1	1	1	0.940207	1
GSM1003593_Kdm2a	1	1	1	0	1
GSM1003594_Kdm2b	0.999991	1	1	0.033931	1
GSM1003596_Rnf2	0.148732	1	1	1	1
GSM1003597_Kdm2b	0.994417	1	1	0.98782	1
GSM1003599_Rnf2	2E-06	1	1	1	1

GSM1003600_Kdm2b	0.942998	1	1	0.999997	1
GSM1019771_Suz12	0	1	0.999523	1	1
GSM1041374_Suz12	0	1	0.999979	1	1
GSM1050291_Sox2	1	0.042059	1	1	0
GSM1050295_Sox2	0.960283	0.731138	0.998235	1	0
GSM1050298_Sox2	0.025236	0.972657	0.969633	0.999419	5.95E-07
GSM1050301_Sox2	0.997975	0.975519	0.999999	1	0
GSM1050304_Sox2	0.479441	0.773446	0.99921	1	0
GSM1059016_Spt5	1	1	1	1.88E-13	1
GSM1059037_Spt5	1	1	1	1.67E-08	1
GSM1082340_Pou5f1	1	0.996475	1	1	0
GSM1082342_Nanog	1	0.999908	1	1	0
GSM1082346_Cdk8	1	0.990808	1	1	0
GSM1082347_Cdk9	1	0.999642	1	1	0
GSM1090228_Tbx3	0.999998	0.999994	0.999984	0.202779	1
GSM1090230_Nanog	1	1	1	0.999976	1
GSM1144547_Tal1	1	1	0.999995	1	1
GSM1169018_Suz12	0	1	0.999962	1	1
GSM1169019_Suz12	0	1	0.989405	1	1
GSM1199188_Suz12	0	1	0.999973	1	1
GSM1246866_Crebbp	1	0.445842	0.999996	1	0
GSM1246868_Thap11	1	0.984452	0.995466	0	1
GSM1359838_Kdm1a	1	0.999999	1	2.48E-06	1
GSM1543575_Tal1	0.999994	0.951684	0.978544	1	1
GSM288347_Sox2	0.256454	0.412826	0.957198	1	0
GSM288348_Smad1	0.418963	0.595561	0.781116	1	0

GSM288350_Tcfcp2l1	1	0.999949	1	1	0
GSM288352_Zfx	1	0.999991	1	9.67E-14	0.995422
GSM288353_Stat3	0.997473	0.04304	0.712866	1	0
GSM288356_Myc	1	0.997893	0.999999	0	1
GSM288360_Suz12	0.784866	0.999157	0.998543	1	1
GSM307138_Sox2	0.940835	0.437865	0.999979	1	0
GSM307142_Tcf3	1.09E-07	0.248632	0.8875	1	0
GSM307144_Suz12	0.998222	0.993052	0.963188	1	1
GSM470523_Nr5a2	1	0.242258	0.999762	1	0
GSM515665_Supt5	1	1	1	0	1
GSM555160_2_TBP	1	0.999705	0.999942	0	1
GSM566280_Wdr5-FL	0.947194	0.985964	0.747896	0.520179	1
GSM590121_Suz12	0	0.999998	0.953436	1	1
GSM590122_Suz12	0	1	0.999599	1	1
GSM590130_Ezh2	0	1	0.981573	1	1
GSM590131_Ezh2	0	1	0.994977	1	1
GSM590132_Ezh2	3.72E-11	0.999993	0.997945	1	1
GSM590133_Ezh2	0	1	0.872485	1	1
GSM611192_Tet1C	2.9E-12	0.999943	0.820324	1	1
GSM611194_Tet1N	0.938095	0.999853	0.999997	4.4E-05	1
GSM647224_p53	1	0.016124	1	1	7.16E-08
GSM647225_p53	1	1	1	1	1
GSM647226_p53S18P	1	8.2E-05	0.998148	1	7.34E-13
GSM647227_p53S18P	1	0.999998	1	1	1
GSM659799_Tet1	0.36798	0.77303	0.972474	0.969895	1
GSM687279_Hdac2	1	0.96413	1	1	0

GSM687280_Hdac2	1	1	0.999904	0	1
GSM687282_Kdm1a	1	1	1	0.360239	0
GSM687284_Chd4	1	0.816681	0.999997	1	0
GSM687285_Rest	1	1	0.999997	1	1
GSM700553_Suz12	0	1	0.999967	1	1
GSM700554_Suz12	0	1	1	1	1
GSM703186_Sox2	0.08814	0.15902	0.995977	1	0
GSM703187_Sox2	0.999999	0.907932	0.999556	1	0
GSM703188_Sox2	0.994038	0.944736	0.976305	1	0
GSM703189_Sox2	0.999987	0.943608	0.993784	1	0
GSM774940_Taf3	1	1	1	0	1
GSM774942_Taf1	1	0.920793	0.984007	1.44E-06	1
GSM774944_Tbp	1	1	1	0	1
GSM788496_Yy1	1	0.999998	1	0.039325	1
GSM896915_Stag2	1	1	1	1	1
GSM896916_Stag1	1	1	1	1	1
GSM935891_Ell3	0.999997	0.191294	1	1	0
GSM937540_Brd4	1	0.971033	1	1	0
GSM937541_Stag1	1	1	1	1	1
GSM937542_Stag2	1	1	1	1	1
GSM972967_Prep	1	0.998682	0.991961	2.46E-09	1
GSM972983_Mbd1a_delMBD	1	0.999997	1	1	0
GSM979714_Tfe3	1	1	0.999943	1	1
GSM979715_Tfe3	1	1	0.999988	1	1

Figure 1: The density plots for binding of RNA polymerase II antibodies, forms and Polycomb components at bivalent enhancers (top) and bivalent promoters (bottom).

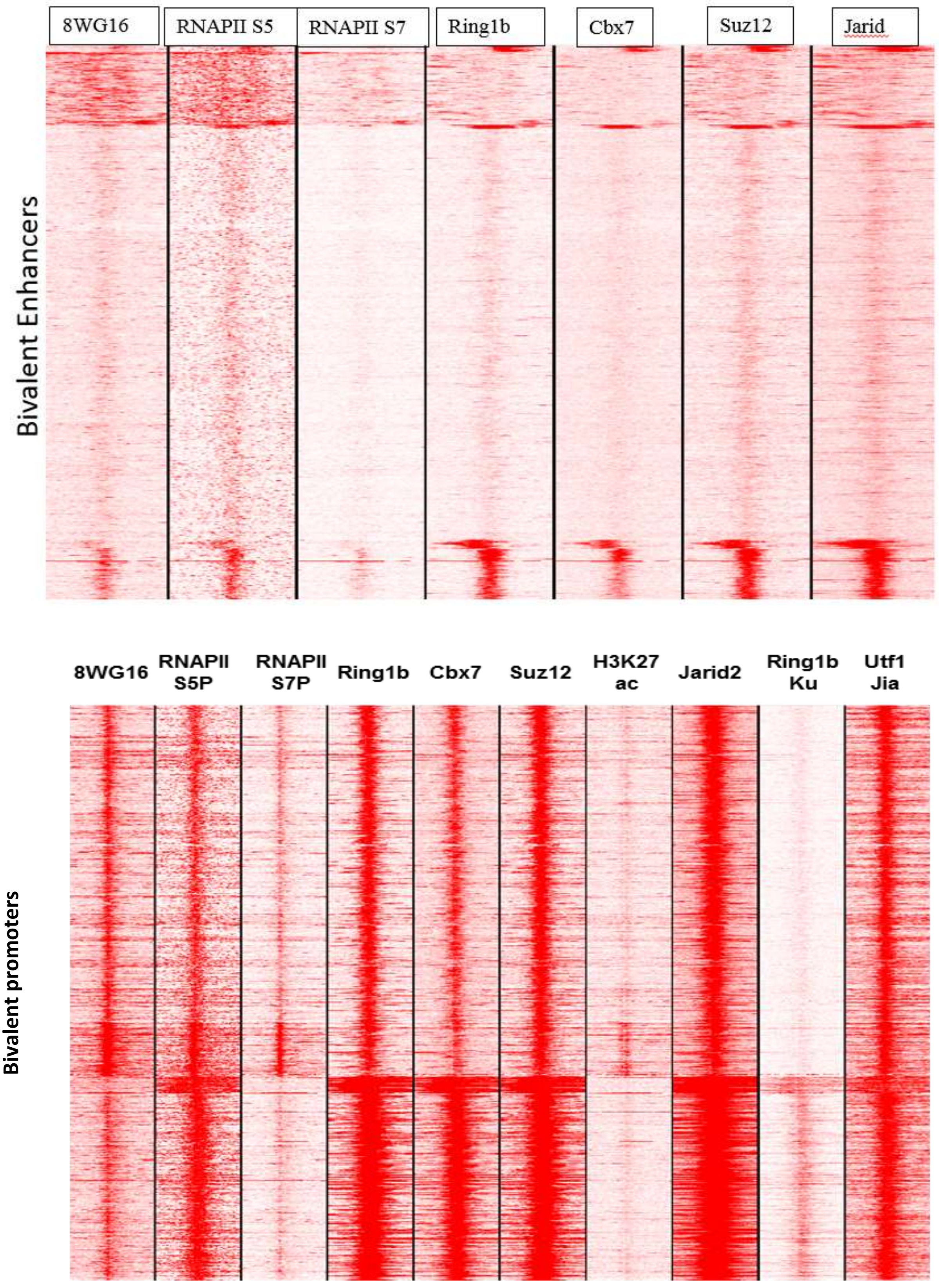

Figure 2: Binding of epigenetic and transcriptional factors enriched at different enhancer groups.

Bivalent enhancers

GSM1014542_Ezh2
GSM566277_Oct4
GSM700556_Phf19
GSM1019771_Suz12
GSM1041374_Suz12
GSM1169018_Suz12
GSM1169019_Suz12
GSM1199188_Suz12
GSM307142_Tcf3
GSM590121_Suz12
GSM590122_Suz12
GSM590130_Ezh2
GSM590131_Ezh2
GSM590133_Ezh2
GSM700554_Suz12

H3K4me1 only enhancers

GSM566278_Rbbp5
GSM651192_Dpy30

H3K27ac only enhancers

GSM1065517_bCatenin
GSM1050291_Sox2
GSM288353_Stat3
GSM647224_p53
GSM647226_p53S18P

H3K27me3 only enhancers

GSM560341_GSM560342_Smc1

Active enhancers

GSM1058995_Kdm4b
GSM1059011_Nanog
GSM1059012_Pou5f1
GSM1059024_Pou5f1
GSM1059031_Pou5f1
GSM1059032_Nanog
GSM1065517_bCatenin
GSM1065518_bCatenin
GSM1184045_Nanog
GSM288345_Nanog
GSM288346_Oct4
GSM288354_Klf4
GSM288355_Esrrb
GSM307137_Oct4
GSM307140_Nanog
GSM560345_Med12
GSM560346_Med12
GSM560347_Med1
GSM566277_Oct4
GSM594600_p300
GSM623989_Prdm14
GSM699164_p300
GSM915092_Ncoa3
GSM1050291_Sox2
GSM1050295_Sox2
GSM1050301_Sox2
GSM1050304_Sox2
GSM1082340_Pou5f1
GSM1082342_Nanog
GSM1082346_Cdk8
GSM1082347_Cdk9
GSM1246866_Crebbp
GSM288347_Sox2
GSM288348_Smad1
GSM288350_Tcfcp2l1
GSM288353_Stat3
GSM307138_Sox2
GSM307142_Tcf3
GSM470523_Nr5a2
GSM687279_Hdac2
GSM687284_Chd4
GSM703186_Sox2
GSM703187_Sox2
GSM703188_Sox2
GSM703189_Sox2
GSM935891_Ell3
GSM937540_Brd4
